# Label-Free LC-MS/MS Proteomics Analyses Reveal Proteomic Changes Accompanying* MSTN* KO in C2C12 Cells

**DOI:** 10.1155/2019/7052456

**Published:** 2019-04-03

**Authors:** Lamei Wang, Yu Huang, Xiaolong Wang, Yulin Chen

**Affiliations:** College of Animal Science and Technology, Northwest A&F University, Yangling 712100, China

## Abstract

Analysis of the proteome of myostatin (*MSTN*) knockout (KO) mouse C2C12 cells has proven valuable to studies investigating the molecular mechanisms by which* MSTN *regulates skeletal muscle development. To identify new protein/pathway alterations and candidate biomarkers for skeletal muscle development, we compared proteomic profiles of* MSTN* KO C2C12 cells (KO) with corresponding wild-type cells (NC) using a label-free liquid chromatography-tandem mass spectrometry (LC-MS/MS) technique. A total of 2637 proteins were identified and quantified in KO cells. Among these proteins, 77 proteins were significantly differentially expressed, 38 upregulated, and 39 downregulated, in* MSTN* KO C2C12 cells. These significantly altered proteins are involved in metabolic processes, developmental processes, immune system processes, and the regulation of other biological processes. Enrichment analysis was utilized to link these alterations to biological pathways, which are predominantly related to oxidative phosphorylation, protein digestion and absorption, mitochondrion localisation, antigen processing and presentation, the MAPK signaling pathway, the PPAR signaling pathway, the PI3K-Akt signaling pathway, and the JAK-STAT signaling pathway. Upregulation of several proteins, including epoxide hydrolase, tropomyosin 1, Cyb5a, HTRA1, Cox6a1, CD109, Synap29, and Ugt1a6, likely enhanced skeletal muscle development, the immune system, and energy metabolism. Collectively, our results present a comprehensive proteomics analysis of* MSTN* KO C2C12 myoblast cells; we hypothesize that* MSTN* KO could activate p38MAPK signaling pathway by CDC42, and we further deciphered the function of* MSTN* in the regulation of skeletal muscle development, immune processes, and mitochondrial energy metabolism.

## 1. Introduction

The myokine MSTN (also termed growth differentiation factor 8, GDF8) is a secreted growth and differentiation factor that belongs to the transforming growth factor-*β* superfamily [1]. Myostatin is a highly conserved negative regulator of skeletal muscle development that controls the proliferation of muscle precursor cells [2, 3] and is mainly expressed in muscles [4]. Several spontaneous mutations in the* MSTN* gene have been found to be correlated with muscle hypertrophy in animals [5] or even in humans [6]; therefore, myostatin dysfunction has been considered a promising strategy for animal breeding or for fighting muscle atrophy in different diseases, including neuromuscular diseases [7]. It has been proven that myostatin can interfere with protein synthesis as well as protein breakdown in proliferating and adult myofibers [8, 9]. Myostatin, along with other TGF-*β*-related factors, also plays key roles in the growth, development, and regulation of diverse cellular functions in other types of cells [10, 11].

Proteins play an important role in many types of molecular networks and perform most of the biochemical functions of living organisms. Intrinsically, cell signal transduction is based on the formation and dissolution of protein-protein interactions, which transmit, translate, and transform stimuli into appropriate biological responses [12]. Significant technological advances over the last decade now allow near exhaustive analysis of the proteomes of various organisms [13–16]. Label-free liquid chromatography-mass spectrometry (LC-MS/MS) routinely quantifies and identifies thousands of proteins across multiple samples in a single run, providing an unprecedented opportunity to examine changes in the proteomics profile of a biological fraction or organism. In addition, gene ontology (GO), Kyoto Encyclopedia of Genes and Genomes (KEGG), and protein-protein interaction (PPI) analysis of proteomics data are conducted to categorize differentially expressed proteins by canonical pathway and molecular function and to identify possible interaction regulators of a protein of interest. Accordingly, in this work, we used a highly sensitive, label-free LC-MS/MS approach to profile and quantify the differential abundance of skeletal muscle proteins in C2C12 myoblasts with* MSTN* gene knockout relative to wild-type cells in order to advance our molecular understanding of myostatin function in skeletal muscle.

Skeletal muscle is a highly specialized tissue that plays a fundamental role in locomotion and is indispensable in regulating whole-body energy metabolism. Recent advances in proteomics and genomic technologies have resulted in significant insights into the biological functions and molecular mechanisms of several proteins and genes.

To date, two studies have examined proteomic changes associated with MSTN dysfunction during pre- and postdevelopment [17, 18]. Chelh et al. found that comparison of protein profiles revealed 20 up- and 18 downregulated proteins spots between* MSTN*-null mice and control littermates [17]. However, this result may reflect the relatively low sensitivity of the two-dimensional gel electrophoresis (2DE) technique utilized. Salzler et al. used high-resolution mass spectrometry coupled with SILAC mouse technology but only quantitated the relative proteomic changes in gastrocnemius muscle from* MSTN* KO mice and mice treated for 2 weeks with REGN1033, an anti-MSTN antibody [18]. However, the MS-based proteomics in this study is more sensitive than 2DE but remains to be improved for detecting changes in low abundant proteins.

In the current study, skeletal muscle C2C12 cells were used to study the molecular mechanisms underlying the biological effects of MSTN from the proteome perspective in order to characterize alterations in the global protein expression of the C2C12 cell proteome in response to MSTN. Cells were labelled, and the proteins were quantified following standard label-free LC-MS/MS proteomics operating procedures. Using label-free LC-MS/MS proteomics, the differentially expressed proteins from* MSTN* KO and normal control group C2C12 cells were accurately characterized, and the authenticity and accuracy of the protein expression detected during the quantitative proteomic examination were further confirmed using Western blotting. The identification of these proteins will facilitate a better understanding of the molecular mechanisms of muscle development underlying MSTN KO. To the best of our knowledge, this is the first gel-less quantitative* MSTN* KO proteome study using label-free mass spectrometry with high mass accuracy in both MS and MS/MS scans.

## 2. Materials and Methods

### 2.1. Cell Culture and Treatments

The mouse skeletal muscle C2C12 myoblast cell line was obtained from the Cell Bank of the Chinese Academy of Sciences (Shanghai, China) and cultured in DMEM/F12 medium (Gibco, UAS) containing 10% FBS (Gibco, UAS), 100 *μ*g/mL streptomycin (Sigma, USA), and 100 U/mL penicillin (Sigma, USA). Cells were seeded at a density of 2 × 10^5^ cells/well in 10-cm plates (Corning, USA) for gene and protein expression studies. Cell cultures were maintained in a humidified 37°C incubator with a 5% CO_2_ atmosphere. The culture medium for the cell lines was replaced every 2 days, and the cells were harvested upon reaching approximately 90% confluence.

The constructs sgRNA1 and sgRNA2, which target the* MSTN* coding region, were designed using the CRISPR Design tool (http://crispr.mit.edu). The paired synthesized oligonucleotides for sgRNAs were annealed and subcloned into the pUC57-U6-sgRNA (Addgene #51132) expression vector. The resulting expression vectors for* MSTN* were confirmed by Sanger sequencing. C2C12 cells were transfected with* MSTN* sgRNA1 (1.5 *μ*g) and* MSTN* sgRNA2 (1.5 *μ*g), along with 1 *μ*g of Cas9 plasmid by Lipofectamine 3000 in a 6-well culture plate. The transfection procedure was carried out using Lipofectamine 3000 Reagent (Invitrogen, USA) according to the manufacturer's instructions. T7 endonuclease I (T7EI) recognizes and cleaves mismatched heteroduplex DNA that arises from the hybridization of wild-type and mutant DNA strands, and cleavage was characterized further by Sanger sequencing. We chose indels at the target sites of* MSTN* skeletal muscle clone cells as treatment groups. For all experiments, empty vector-treated C2C12 cells were used as controls, and 3 independent experiments were performed as biological replicates. Off-target analysis was performed using a bioinformatics-based search tool (Cas-OFFinder) to select potential off-target sites, which were evaluated using Sanger sequencing to confirm gene modification frequencies for the CRISPR/Cas9 system. We chose indels at the target sites of* MSTN* skeletal muscle clone cells as treatment groups. For all experiments, empty vector-treated C2C12 cells were used as controls, and 3 independent experiments were performed as biological replicates.

### 2.2. RNA Extraction and Gene Expression Analysis

Total cellular RNA was isolated from the samples using an RNA extraction kit (Promega, Beijing, China), following the manufacturer's instructions. Two micrograms of total RNA was transcribed into cDNA using an Invitrogen SuperScript™ One-step RT-PCR Kit. A real-time quantitative PCR assay was carried out with SYBR Premix Ex Taq II (Takara, China) and monitored with a CFX96 Touch Real-time PCR Detection System (Bio-Rad, USA). *β*-actin served as the reporter gene. All primer sequences used in this assay are shown in Supplementary Information Table S1. The expression data were analyzed using the 2^-ΔΔCT^ method.

### 2.3. Sample Preparation and Protein Digestion for a Label-Free Experiment

For label-free experiments, proteins were extracted from the samples of both control and* MSTN* KO cells in triplicate. The cell pellets were washed twice with cold PBS. For label-free experiments, 200 *μ*L of cold SDT lysis buffer (4% SDS, 100 mM DTT, 150 mM Tris-HCl at pH 8.0) was added to the cell pellets on ice; afterward, the samples were disrupted by agitation using a homogenizer (Fastprep-24 ®, MP Biomedical) and boiling for 10 min. The samples were further ultrasonicated and incubated in boiling water for another 5 min, and the undissolved cellular debris was removed by centrifugation at 14,000 g for 30 min at 4°C. Protein concentrations of BCA determined cell lysates were adjusted to 200 *μ*L of each sample. The sample was stored at -80°C.

### 2.4. SDS-PAGE Separation

The 5 x loading buffer was used to mix with the 20 *μ*g proteins of each sample respectively and the mixture was boiled for 5 min. The proteins were separated on 12.5% SDS-PAGE gel (constant current 14 mA, 120 min). Coomassie Blue R-250 staining was used to visualize protein bands.

### 2.5. Protein Digestion

Approximately 250 *μ*g of protein from each sample was digested using the filter-aided sample preparation (FASP) method as previously described [19]. Put it in simple, a protein sample was suspended in UA buffer (8 M urea, 150 mM Tris-HCl, pH 8.0) to removed detergent, DTT and other low-molecular-weight components by repeated ultrafiltration (Microcon units, 10 kD). After ultrafiltration, to block reduced the cysteine residues, the 100 *μ*L of iodoacetamide (100 mM IAA in UA buffer) was added and incubated for 30 min in the dark. Next, the filter was washed with 100 *μ*L of UA buffer at 14,000 g for 10 min and twice with 0.025 M (100 *μ*L) ammonium bicarbonate. Then, the protein samples were added to 100 *μ*L of trypsin stock solution (8 *μ*g of trypsin in 100 *μ*L of NH_4_HCO_3_) for digested proteins by gentle overtaxing for 20 s and incubated at 37°C for 16 h. The digested peptides in each sample were desalted on C18 Cartridges (Empore™ SPE Cartridges C18 (standard density), bed I.D. 7 mm, volume 3 mL; Sigma) and then concentrated by centrifugation at 14,000 g for 10 min and were reconstituted in 50 *μ*L of 0.1% (v/v) trifluoroacetic acid. Based on the frequency calculation of tryptophan and tyrosine in vertebrate proteins, the peptide content was quantified by using extinction coefficient of 1.1 with 0.1% (wt/vol) solution under UV light spectral density at 280 nm.

### 2.6. LC-MS/MS Analysis

The LC-MS/MS analysis was conducted on an Easy nLC Liquid Chromatograph (Thermo Fisher Scientific) coupled to a Q Exactive mass spectrometer (Thermo Fisher Scientific) for 120 min. Next, the peptides were desalted on C18 Cartridges (Empore™ SPE Cartridges C18, bed I.D. 7 mm, volume 3 mL, Sigma), concentrated by vacuum centrifugation and reconstituted in 40 *μ*L of 0.1% (v/v) formic acid. Peptides were separated on a C18-reversed phase analytical column (Thermo Scientific Easy Column; 10 cm length, 75 *μ*m inner diameter, 3-*μ*m resin) over a 120 min gradient from buffer A (2% acetonitrile and 0.1% formic acid, vol/vol) and B linear gradient solvent (84% acetonitrile and 0.1% formic acid) at a flow rate of 250 nL/min controlled by IntelliFlow technology. Data-dependent acquisition was performed with MS scan mass window set at 300−1800 m/z, and top 10 charge state ions were selected for fragmentation. Dynamic exclusion time was set to 50 s. Survey scans were acquired at 17,500 with a maximum ion injection time at 200 m/z. The normalized collision energy was 30 EV, and the underfill ratio, which specifies the minimum percentage of the target value likely to be reached at the maximum fill time, was defined as 0.1%. The instrument was run with the peptide recognition mode enabled. Each sample was analyzed in triplicate.

### 2.7. Data Calculation and Analysis

The raw LC-MS/MS data from all samples were analyzed using MaxQuant software (http://maxquant.org/, version 1.5.3.17) [20, 21] and searched against UniProt_mouse_83374_20170829 and FASTA databases (83374 total entries, downloaded 08/29/17) using the built in Andromeda search engine [22]. The database patterns were shown using Target-Reverse. The following search parameters were set: enzyme: trypsin, maximum miss cleavage: 2, precursor mass window: 6 ppm, precursor mass tolerance: 20 ppm, fixed modification: Carbamidomethylation of cysteines, variable modifications: protein N-terminal acetylation and methionine oxidation. Statistical analysis that compared the difference between the* MSTN* KO and NC groups was performed by unpaired t-test. Differential proteins were screened with the following criteria: target FDR (strict) was set as 0.01 and target FDR (relaxed) was set as 0.05. The identification of the protein was allowed with a maximum 1% false positive discovery rate in at least three technical replicate injections.

Label-free quantification was carried out in MaxQuant software as previously described [23]. Protein abundance was calculated using normalized spectral protein intensity (LFQ intensity). The LFQ intensity values were Log_2_ transformed, and missing values were imputed with random numbers from a normal distribution.

### 2.8. Bioinformatics Analysis

Hierarchical clustering analysis was performed using Cluster 3.0 (http://bonsai.hgc.jp/~mdehoon/software/cluster/software.htm) and the Java Treeview software (http://jtreeview.sourceforge.net) for protein relative expression data. Meanwhile, hierarchical clustering analysis was further processed using Euclidean distance algorithm for similarity measure and average linkage clustering algorithm (clustering uses the centroids of the observations). Heatmap is often presented as a visual aid in addition to the dendrogram.

The user-defined search parameters in InterProScan were used for the GO annotation: the top 10 blast hits with E-value less than 1*e*-3 for each query sequence were retrieved and loaded into* Blast2GO*_1_ (Version 3.3.5) for GO_2_ mapping and annotation. An annotation configuration with a filter of 1*e*-6 E-value was selected, with the default gradual EC weights, a GO weight of 5, and an annotation cutoff of 75 was chosen. Unannotated sequences were then reannotated with more permissive parameters. The sequences without BLAST hits and unannotated sequences were then selected to compare with an InterProScan_3_ and EBI databases to retrieve functional annotations of protein motifs and merge the InterProScan GO terms to the annotation set. The GO annotation results were plotted by R scripts.

Database enrichment analysis was performed against the UniProtKB database (Release 2016_10) in FASTA format. Mus musculus was chosen as the organism for enrichment analysis. The obtained peptide/protein list was exported to Microsoft Excel for further analysis. In this work, these proteins were further subjected to EBI databases to find GO annotations. Online available databases: UniProt (http://www.uniprot.org), KEGG (Kyoto Encyclopedia of Genes and Genomes) (http://www.genome.jp/kegg/), and NCBI (National Center for Biotechnology Information) (https://www.ncbi.nlm.nih.gov/) were used for the GO and KEGG. GO enrichment on three ontologies (biological process, BP, molecular function, MF, and cellular component, CC) and KEGG pathway enrichment analyses were applied based on the Fisher' exact test, considering the whole quantified protein/phosphoproteins annotation as background dataset. Benjamini- Hochberg correction for multiple testing was further applied to adjust derived* p*-values. And only functional categories and pathways with* p*-values < 0.05 were considered as significant.

### 2.9. Protein-Protein Interaction (PPI) Analysis

PPIs were studied using the IntAct molecular interaction database (http://www.ebi.ac.uk/intact/) according to gene symbols or STRING software (http://string-db.org/). Furthermore, the degree of each protein was calculated to evaluate the importance of the protein in the PPI network. The results were downloaded in the XGMML format and imported into Cytoscape56 software (http://www.cytoscape.org/, version 3.2.1) to visualize and further analyze functional protein-protein interaction networks. Furthermore, the degree of each protein was calculated to evaluate the importance of the protein in the PPI network.

### 2.10. Western Blotting

To validate our label-free LC-MS results, Western blot analyses were performed on whole myoblast C2C12 cell protein extracts. Cells were lysed in ice-cold RIPA buffer (Beyotime, China) in the presence of protease inhibitors and 1% DTT; the extracts were centrifuged at 12,000 rpm for 20 min at 4°C, and the supernatants were transferred to new Eppendorf tubes. The total protein concentration was determined using the Pierce BCA Protein Assay Kit (Thermo Fisher Scientific, USA) according to the manufacturer's instructions. Total protein extracts (every 20 *μ*g) were separated on 10% or 12% SDS-PAGE gels and then were transferred to PVDF membranes (Roche, USA) using a semidry transfer blotter. The membranes were blocked with 5% skimmed milk powder in TBST for 1 h and then were incubated with primary polyclonal antibody (anti-GDF8, Santa Cruz, USA; Anilin, Santa Cruz, USA; UGT1a6, Abcam, UK; Cox6b1, Santa Cruz, USA; Tgfb1i1, Santa Cruz, USA) overnight at 4°C. After washing three times, the membranes were incubated at room temperature for 1-2 h with horseradish peroxidase (HRP)-conjugated goat anti-rabbit IgG (Beyotime, China) or mouse IgG*κ* binding protein-HRP (Santa Cruz, USA) secondary antibody. Detection was performed using chemiluminescence luminal reagents (Millipore, USA).

### 2.11. Statistical Analyses

Data were analyzed using GraphPad Prism 7 Software (GraphPad Software, La Jolla, CA). For statistical analysis, one-way ANOVA with Tukey's post hoc test or a two-tailed Student's t-test was used to determine the significance. The data are shown as the mean ± SE.* P* values ≤ 0.05 were considered significant.

## 3. Results

### 3.1. Successful Generation of CRISPR/Cas9-Mediated MSTN Knockout in C2C12 Myoblasts

To ensure the success of mutagenesis by CRISPR/Cas9, sgRNA1 and sgRNA2 were synthesized to target the functional domain in exon 3. The T7EI mutation detection assay was performed to detect the mutational efficacy of* MSTN*. The PCR products from C2C12 myoblasts in the control group (empty vector-treated C2C12 cells) showed one distinct band (725 bp), while those from* MSTN* KO myoblasts showed two or more bands (725 bp). Gel electrophoresis revealed various banding patterns depending on the type of mutation (Supplementary Figure S1A). Because the efficacy of* MSTN* sgRNA1 knockout was much higher than that of* MSTN* sgRNA2, we chose* MSTN* sgRNA1 knockout cells for Sanger sequencing. Cleavage was confirmed by sequence analysis, which showed that there was a 2-bp deletion in the* MSTN* gene target site of clone number 3. The 2-bp deletion occurred at position 859-860 with respect to the ATG start codon. The number 11 cell clone contained a 4-bp deletion between 857 and 867, whereas the number 20 cell clone contained a 1-bp deletion at position 860. The observed indels at the target sites of* MSTN* with a range of mutation sizes are shown in Supplementary Figure S1B. We also assessed the off-target activity of the mutant cells by sequencing predicted off-target sites for both gRNAs, and no mutation was detected (Supplementary Figure S2).

As Supplementary Figure S3A shows, we found morphology of* MSTN* KO C2C12 cells was significantly bigger than NC C2C12 cells from the photo of a microscope. We found lower expression levels of the* MSTN* gene in* MSTN* KO C2C12 myoblasts than in the control group (Supplementary Figure S3B). Consistent with the qPCR analysis, the Western blotting analysis showed lower levels of* MSTN* protein in* MSTN* KO cells than in control cells (Supplementary Figure S3C).

### 3.2. Identification and Quantification of Proteins from MSTN KO and Control Cells

**Figure 1:**
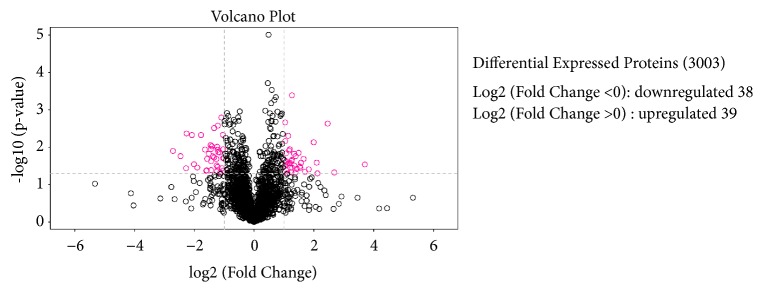
Mass spectrometry-based quantitative proteomic analysis of skeletal muscle C2C12 cell proteins. Volcano plot of a total of 3003 quantified proteins illustrating increased and decreased expression following* MSTN* knockout. The horizontal coordinate is the difference multiple (logarithmic transformation at the base of 2), and the vertical coordinate is the significant difference p value (logarithmic transformation at the base of 10). Red dots represent the expression profiles of 77 significantly different proteins showing a fold change ≥ ± 2 in their relative abundance in* MSTN* KO cells relative to control cells.

**Figure 2:**
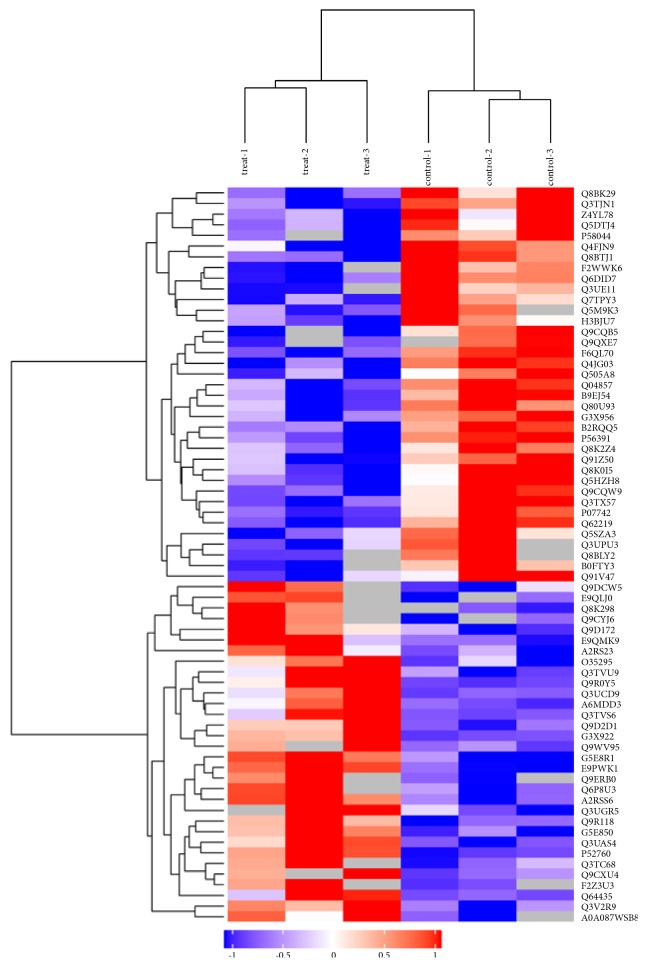
A hierarchically clustered heatmap of the relative abundances of proteins in the two sample types under study, shown as the expression patterns of the top 69 DEPs. The red blocks represent the overexpressed proteins, and the blue blocks represent proteins with the lowest expression levels. Coloured bars indicate the expression levels.

The protein profiles, analyzed by MaxQuant 1.5.3.17, of* MSTN*-knockout C2C12 cells and control cells were compared. We used a false discovery rate (FDR) ≤ 0.01 as the threshold to judge the significance of differences in protein expression. The label-free LC-MS/MS results indicated that the* MSTN* KO cells yielded 2,637 proteins, whereas the control cells yielded 2,779 proteins; among these, 2,413 proteins shared common datasets. Statistical analysis of the expression of these proteins revealed significant changes in 77 associated proteins in the* MSTN-*knockout cells with > 2-fold changes relative to control cells: 39 proteins were upregulated, whereas 38 proteins were downregulated. Volcano plots of the DEPs are shown in Figure 1. Hierarchical clustering provided the expression profiles of the top 69 DEGs (Figure 2). To further seek the target proteins related to muscle development. We found that mitochondrial, CDC42 was changed in DEPs. As well known, skeletal muscle development was regulated by CDC42 [24]. The top 20 significantly upregulated or downregulated proteins from KO cells relative to NC cells are summarized in Supplementary Table S1.

### 3.3. Functional Classification Annotation Analysis of Differentially Expressed Proteins

**Figure 3:**
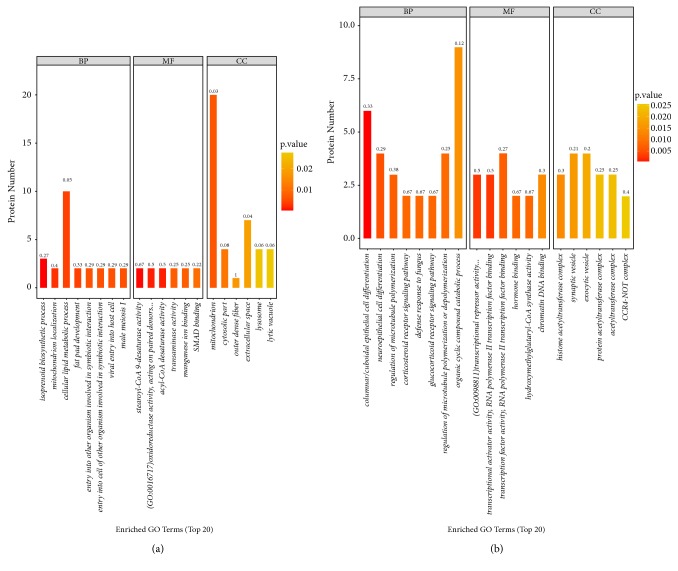
Gene Ontology (GO) annotation of the 20 most differentially accumulated proteins (DAPs) in C2C12 cells under* MSTN*-knockout conditions. (a) KO versus NC, analysis of significantly different proteins; (b) KO versus NC, analysis of present or absent proteins.

**Figure 4:**
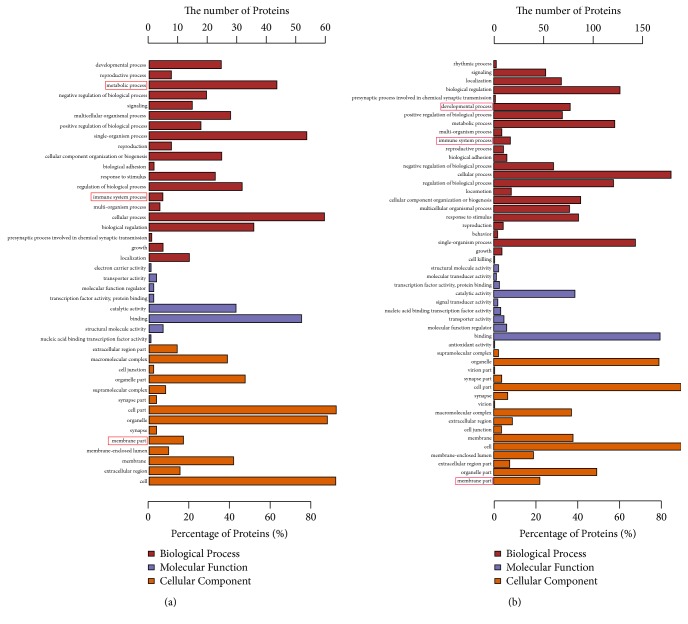
Level-two gene ontology (GO) annotation of differentially accumulated proteins (DAPS) in C2C12 cells. Classification of the annotated amino acid sequences. Amino acid sequences were grouped into different functional subcategories: cellular component (CC), molecular function (MF), and biological process (BF). (a) KO versus NC, analysis of significantly different proteins; (b) KO versus NC, analysis of present or absent proteins. Figure red dotted portions represent cell development-relative and immune system GO terms.

To further investigate* MSTN* KO C2C12 skeletal muscle cells, GO analysis was performed to generate classification clusters based on biological processes, cellular components, and molecular functioning. The top 20 enriched GO terms are shown in Figure 3. By comparing the protein list from* MSTN* KO cells with that of the NC group, major GO terms were found to be relevant to mitochondrion localisation, stearoyl-CoA 9-desaturase activity, SMAD binding, lysosome, neuroepithelial cell differentiation, immune system process, and developmental process. Furthermore, level-two GO terms relevant to synapse part, lysosome, microtubule cytoskeleton, actin cytoskeleton, cytoskeleton, mitochondrial outer membrane, mitochondrial envelope, and the nucleus were found to be enriched in the CC. In terms of molecular function, electron carrier, transporter activity, molecular function regulator, transcription factor activity, protein binding, catalytic activity, binding, structural molecule activity, and nucleic acid binding transcription factor activity were found to be enriched. Moreover, immune system process, developmental process, regulation of transforming growth factor beta receptor signaling pathway, cell cycle process, and metabolic process were significantly enriched within BP. As shown in Figure 4, we found that the cell cycle process contains the protein CDC42, and MAPK signaling pathway contains the protein MAPK3.

### 3.4. Pathway Analysis

**Figure 5:**
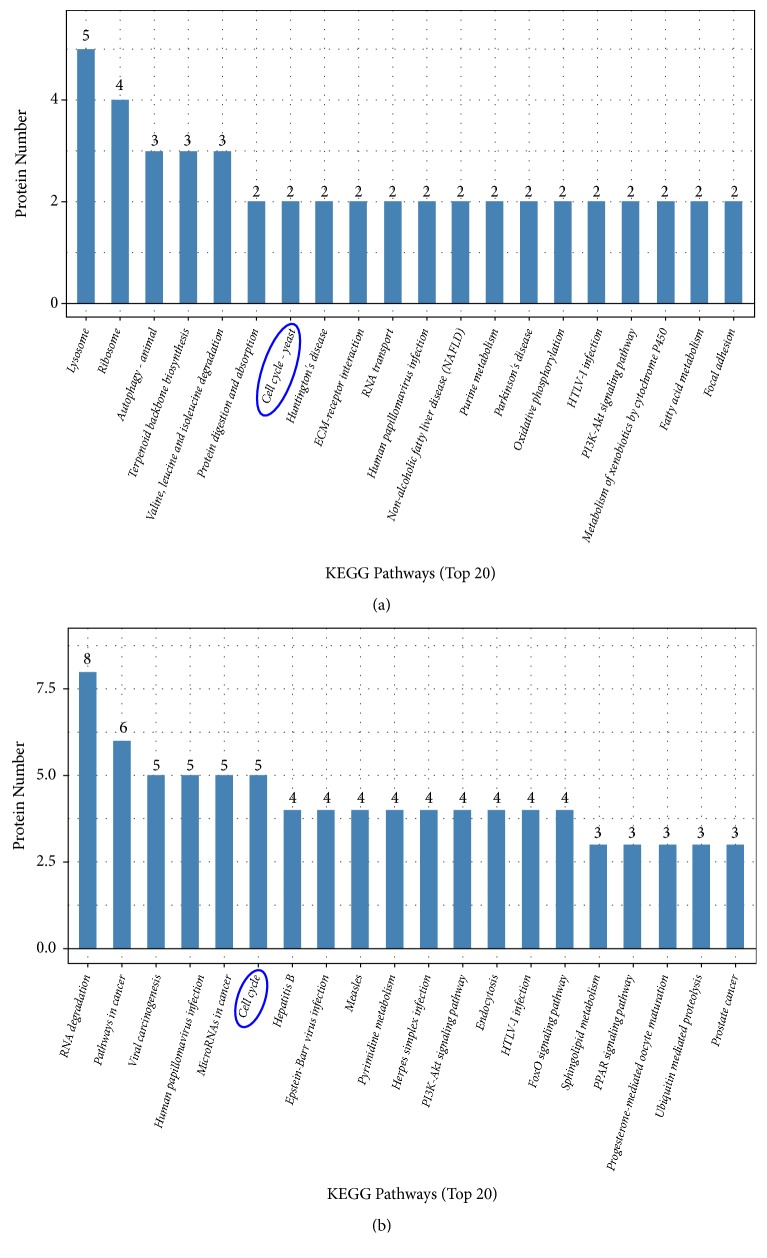
Top 20 KEGG pathways enrichment analysis. (a) KO versus NC, analysis of significantly different proteins; (b) KO versus NC, analysis of present or absent proteins. Figure blue dotted portions represent cell development-relative KEGG pathway.

**Figure 6:**
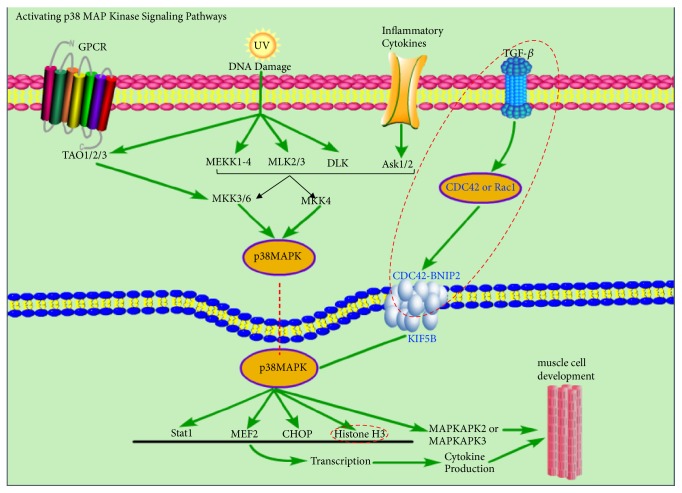
Activating p38 MAPK intersection signaling pathway. Figure red dotted portions represent differential proteins.

Specific protein functions should also be reflected by pathway information. We mapped all identified proteins to the KEGG pathway database. Statistical enrichment of the 77 significantly altered proteins (KO versus NC) was calculated for each KEGG pathway. The top 20 pathways were identified by comparing the complete list of proteins with significantly different abundance and proteins that were present or absent in KO and NC cells. The results from the KEGG analysis revealed that many proteins related to alpha-linolenic acid metabolism (delta-6 desaturase), the PPAR signaling pathway (delta-6 desaturase), antigen processing and presentation (cathepsin B), and the MAPK signaling pathway (F-box-like/WD repeat-containing protein TBL1X, MAPK3) were also enriched (Figure 5). In the current study, we found that CDC42 was changed by* MSTN* KO, activating p38 MAP Kinase signaling pathway as shown in Figure 6.

In addition, several proteins were typically enriched in oxidative phosphorylation, the FOXO signaling pathways, the PPAR signaling pathway, the PI3K-AKT signaling pathway and the JAK-STAT signaling pathway, as shown in Supplementary Figures S4 and S5. Proteins associated with skeletal muscle cell development, fatty acid metabolism, the immune system and mitochondrial energy metabolism.

### 3.5. Protein-Protein Interaction (PPI) Analysis

**Figure 7:**
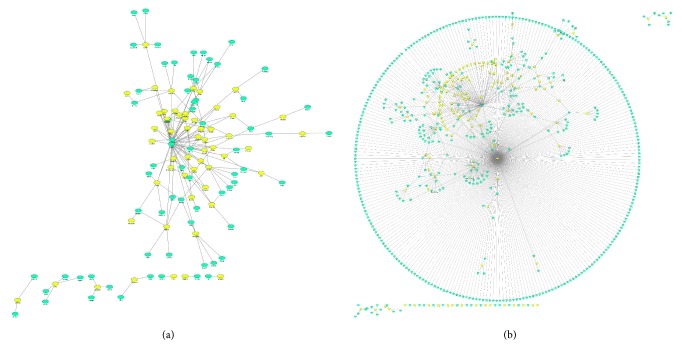
Differentially expressed proteins of KO relative to NC cells, as depicted in their networks by STRING and visualized by Cytoscape5 software. Each yellow dot represents a protein of interest found to be differentially expressed in the presence of* MSTN* KO by quantitative proteomics, and the lines represent putative protein interactions recorded or predicted by STRING. Green dots represent proteins of interest directly interacting with other proteins. (a) KO versus NC, analysis of significantly different proteins; (b) KO versus NC, analysis of present or absent proteins.

Ingenuity analysis of interaction networks of skeletal muscle cell proteins in C2C12 myoblasts was performed as part of the PPI analysis. Proteomics regulation events were analyzed by using STRING software to map the 69 proteins with significant changes and 209 proteins that appeared or disappeared between the KO and NC cells in their direct PPI networks (Figure 7).

### 3.6. Western Blot Analysis

**Figure 8:**
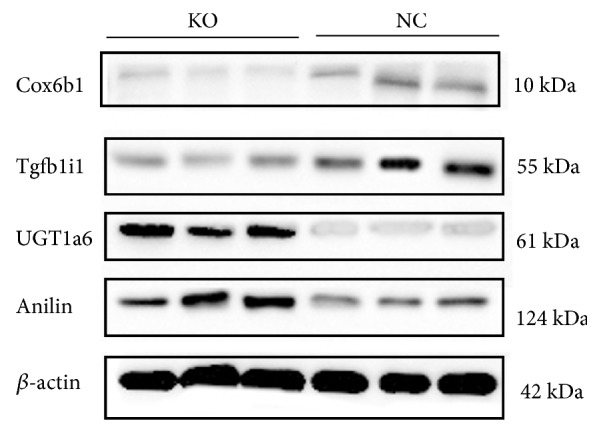
Western blot analysis and immunofluorescence analysis of the Cox6b1, Tgfb1i1, UGT1a6, and Anilin proteins. *β*-actin (loading control) demonstrated equivalent protein loads in each lane. KO:* MSTN* KO clone cell lines. NC: normal control group.

To validate the outcomes of label-free analysis at the proteomic level, we performed Western blotting using the cell protein extract to assess key protein content that might be involved in muscle development and other physiological processes. Anilin, UGT1a6, Cox6b1, and Tgfb1i1 were selected for further confirmation of the LC-MS/MS proteomics results based on differences in signaling pathways, molecular localisation, and the novelty of biological function. Up- and downregulation of proteins were confirmed by Western blot analysis. Immunoblotting revealed that Anilin and UGT1a6 showed significant upregulation in terms of protein expression, and the Cox6b1 and Tgfb1i1 proteins showed significant downregulation in C2C12 cell samples. Overall, the Western blot results showed that the LC−MS/MS proteomics data were reliable and accurate (Figure 8).

## 4. Discussion

Proteomic changes relating to* MSTN* gene mutation have been studied, and the results vary depending on experimental models. In our previous studies, we used RNA-seq to reveal the transcriptome profile in myostatin gene-knockout goats [25]. Salzler et al., using high-resolution mass spectrometry coupled with SILAC mouse technology, quantitated the relative proteomics changes in gastrocnemius muscle from* MSTN*-knockout (*MSTN*^−/−^) mice and mice treated for 2 weeks with REGN1033, an anti-*MSTN* antibody. Functional annotation of the altered proteins in* MSTN*^−/−^ mice corroborated multiple physiological changes, including the slow-to-fast fiber type switch [18]. Puddick et al. used a comparative proteomic method to quantify proteins change in skeletal muscle mitochondria from MSTN-null mice [26]. It is well established that myostatin is mainly expressed in skeletal muscles and potently inhibits skeletal muscle development [27]. Since its discovery, many studies have demonstrated the mechanism by which myostatin promotes the loss of protein in skeletal muscles [28–30]. Despite this very clear phenomenon, some conflicting evidence remains concerning biological processes that are altered in the presence of myostatin. Differences may be due to the different concentrations of myostatin used, the myostatin isoform (active homodimer or full-length), and methodologies [31].

Here, we used* MSTN* gene knockout cells to investigate the differential expression of proteins and demonstrated that 39 proteins were significantly upregulated, while 38 proteins were downregulated, with changes greater than a 2-fold difference. Further, we found a total of 3003 peptides and proteins in the KO cells that met the criterion of a global false discovery rate cutoff of<1% based on the Andromeda search engine. In the present study, several of the identified DEPs are typically associated with skeletal muscle cell development, including Fst1, CD109, transforming growth factor *β*-1-induced transcript 1 protein (Tgfb1i1) and HTRA1. Fst1 is a secreted glycoprotein that was first identified as a potent inhibitor of some members of the TGF-*β* superfamily because of its strong binding affinity for the receptor protein activin. Fst1 can block the activity of myostatin via competitive binding [32]. CD109 is a glycosylphosphatidylinositol-anchored glycoprotein that negatively regulates the TGF-*β* signaling pathway. CD109 promotes TGF-*β* receptor I internalization and degradation by regulating SMAD7 and Smurf2 activities, which inhibit the TGF-*β* signaling pathway [33]. Tgfb1i1, also known as hydrogen peroxide-inducible clone-5 (Hic-5), was found to be induced by TGF-*β* and is a focal adhesion scaffold LIM-containing protein with homology to paxillin [34]. It was reported that Tgfbli1 upregulates TGF-*β* signaling through its ability to directly interact with and neutralize Smad7 in a myofibroblast cell line [35]. HTRA1 is a member of the high-temperature requirement A (HTRA) family of serine proteases. Mammalian HTRA1 plays a role in a variety of normal physiological processes, including protein degradation and cell signaling, and has been implicated in skeletal development and osteogenesis [36, 37]. Mutations in the* HTRA1* gene can decrease HTRA1 protease activity, consequently leading to the disinhibition of TGF-*β* family signaling. TGF-*β* plays a key role in bone remodelling by inducing osteoblast differentiation and proliferation [38]. Up to now, there has been no study investigating the interactive relationships among Fst1, CD109, Tgfb1i1 and HTRA1 and MSTN. The interactions may be to be a new mechanism for* MSTN* to regulate muscle development.

*MSTN* dysfunction may lead to protein changes in biological processes. Several DEPs are immune system-related proteins, including interferon-induced transmembrane protein 3 (IFITM3), Rpl39, Mcl-1 ubiquitin ligase, CD109 antigen and, Anilin. IFITM3 is a cellular restriction factor that inhibits infection by the influenza virus and many other pathogenic viruses. IFITM3 prevents endocytosed virus particles from accessing the host cytoplasm. IFITM3 modulation of endocytic compartments and posttranslational regulation may also be important for other potential functions of IFITM isoforms as well as immune evasion by pathogens [39]. We showed that IFITM3 (Q9CQW9) was downregulated in* MSTN* KO cells. However, Anilin was upregulated in* MSTN* KO cells. Anilin is a 124-kDa protein that is highly concentrated in the cleavage furrow in numerous animal cells with a pattern that resembles that of Rho A [40]. Rho A promotes nucleation, elongation, and sliding of actin filaments through the coordinated activation of both formin proteins and myosin II motors [41]. Meanwhile, Anilin is required for cytokinesis as an essential component for the structural integrity of the cleavage furrow and, for the completion of cleavage furrow ingression, plays a role in bleb assembly during metaphase and anaphase of mitosis, and likely functions in podocyte cell migration. Mcl-1 is an antiapoptotic protein of the Bcl-2 family that is essential for the survival of multiple cell lineages and is highly amplified in human cancer development. Under physiological conditions, Mcl-1 expression is tightly regulated at multiple levels, involving transcriptional, posttranscriptional, and posttranslational processes. Ubiquitination of Mcl-1, which targets it for proteasomal degradation, allows for the rapid elimination of the protein and triggering of cell death in response to various cellular events [42]. A previous study demonstrated that myostatin may influence immune cell development in mammals [43]. Additionally, we found that the JAK-STAT signaling pathway was significantly changed in* MSTN* KO cells. JAK-STAT signaling is essential for antiviral immunity, making INF-*α* an obvious antiviral therapeutic [44]. This study is in accordance with the previous studies described above showing that myostatin gene knockout changes the immune system in C2C12 cells.

The lysosomal, Ca^2+^-dependent, and ubiquitin-proteasome (UPS) systems are considered to be the three main proteolytic processes involved in the control of muscle protein metabolism in mammals [31]. The lysosome plays an essential role in sensing and signaling cellular nutrient status by recruiting MTORC1 (mechanistic target of rapamycin complex 1), a ubiquitous protein kinase acting as a key regulator of autophagy. MTORC1 is stimulated by amino acids through an inside-out mechanism in which amino acids must accumulate in the lysosome lumen to initiate signaling [45].

Proteomic changes relating to* MSTN* gene mutation have been studied, and the results vary depending on experimental models. In our previous studies, we used RNA-seq to reveal the transcriptome profile in myostatin gene-knockout goats [25]. Salzler et al., using high-resolution mass spectrometry coupled with SILAC mouse technology, quantitated the relative proteomics changes in gastrocnemius muscle from* MSTN*-knockout (*MSTN*^−/−^) mice and mice treated for 2 weeks with REGN1033, an anti-*MSTN* antibody. Functional annotation of the altered proteins in* MSTN*^−/−^ mice corroborated multiple physiological changes, including the slow-to-fast fiber type switch [18]. Puddick et al. used a comparative proteomic method to quantify proteins change in skeletal muscle mitochondria from* MSTN*-null mice [26]. In this study, we compared the proteomic changes between myostatin-null myoblasts and wild-type myoblasts and found profound changes in cellular protein expression profiles. These relevant researches intercomparing are shown in the Supplementary Table S3. Most mitochondrial pathways, including oxidative phosphorylation and the TCA cycle, were significantly changed; Cox6b, Cyb5a, Cox6a1, isocitrate dehydrogenase, succinate dehydrogenase, ATP synthase subunit alpha, and malate dehydrogenase were all upregulated. We showed that the oxidative phosphorylation pathway and immune system processes both include Cox6a1. In a previous report, Cox6a1 was shown to play a role in energy metabolism, and recent reports have suggested that Cox6a1 suppresses Bax- and 4-HPR-mediated cell death and inhibits ROS production. Cox6a1 is also involved in stress-induced apoptosis and neurodegenerative diseases in organs with a high-energy demand [46–48]. We showed that knockout of* MSTN* could increase the expression of Cox6a1 in C2C12 cells. Therefore,* MSTN* has a role in mitochondrial energy metabolism.

These DEPs are involved in many biological processes, including isoprenoid biosynthetic process, cellular lipid metabolic process, immune system process, synaptic process involved in chemical synaptic transmission, Wnt signaling pathway, ribosome biogenesis, skeletal system morphogenesis and the MAPK signaling pathway, and several proteins were involved in the mitochondrial oxidative phosphorylation pathway. Ribosome biogenesis in eukaryotes, the spliceosome and mRNA surveillance pathways are central processes for gene expression and protein synthesis, which are inextricably associated with cell growth and division [49]. Kang et al. reported that the p38MAPK pathway promotes skeletal myogenesis; the Cdo-BNIP2 interaction stimulates CDC42 activity, which in turn promotes p38*α*/*β* activity and cell differentiation [24]. In current study, MSTN dysfunction leads to changes in CDC42 activity, with these results are likely to reveal previous unknown mechanisms between* MSTN* and downstream modulation of CDC42-BNIP2 activity, during myoblast differentiation.

To provide an efficient way to illustrate the molecular mechanisms of protein expression in C2C12 cells harbouring the knockout* MSTN* gene, we obtained protein-protein interaction (PPI) information from the online STRING 10 database. The central nodes in directed and undirected PPI networks show individual proteins, and the lines represent their relationships. The nodes of DEP-derived interaction networks were determined using the degree and combined score between two nodes. In addition to the networks described above, Ywhae, Hsd3b4, Cox6a1, Tpm1, Stat, Mad211, and Ctsd in PPI networks were found to represent major hubs. Additionally, this program also indicated that the same proteins might be involved in many biological activities, such as fatty acid metabolism, the biosynthesis of unsaturated fatty acids, the PPAR signaling pathway, and *α*-linoleic acid metabolism.

## 5. Conclusion

In this study, we performed a label-free quantitative proteomics technique by using LC-MS/MS to analyze* MSTN* KO C2C12 cell lines. A total of 3003 unique proteins were detected and quantified in our study, providing a database for quantified proteomics in C2C12 cells during the proliferation and differentiation phases. Integrated analysis of the proteome data revealed ten genes (Tpm1, HTRA1, Fads, Myf9, Ckap5, Fst1, Tim23, PAK2, Rho A, and CD109). Importantly, the HTRA1 and Fst1 genes were reported to be strong candidates for mediating TGF-*β* signaling pathway regulation. Direct protein-protein interaction network analyses and KEGG pathway mapping revealed that muscle mitochondrial energy metabolism, immune system processes and development were mediated by* MSTN* in C2C12 cells. Meanwhile, MSTN dysfunction possibly activate p38 MAPK signaling pathway by CDC42-BNIP2. Our data provide valuable insights into the role of the myostatin protein in muscle development, immune system processes, and energy metabolism in skeletal muscle for future studies.

## Data Availability

The figures and tables data used to support the findings of this study are included within the supplementary information files 1 and 2.

## Abbreviations

DEGs: Differentially expressed genes
DSG: Desmoglein
DSC: Desmocollin
DSG 3: Desmoglein 3
FDR: False discovery rate
HCD: High-energy collisional dissociation
iTRAQ: Isobaric tag for relative and absolute quantitation
KAPs: Keratin-associated proteins
KO: Knockout
LC-MS/MS: Liquid chromatography-tandem mass spectrometry
PTM: Posttranslational modification
PCA: Principal-component analysis
PC1: Principal component 1
SCX: Strong cationic-exchange chromatography.

## Supplementary Materials

Supplementary Materials Supplementary Figure S1: detection of MSTN CRISPR/Cas9-mediated targeting in C2C12 cells. (A) Mutation detection in C2C12 cells by T7EI cleavage assay. #3, #11, and #20 represent different MSTN KO cell clones. M: marker; WT: wild-type PCR products from C2C12 cells that were not treated with CRISPR/Cas9. (B) Sequences of modified MSTN alleles. Insertions are in red; deletions (-), insertions (+) shown to the right of each allele. Supplementary Figure S2: analysis of MSTN gene and protein expression in C2C12 myoblasts. (A) Relative expression level of MSTN was determined by qRT-PCR. (B) Western blot analysis of myostatin protein from NC and MSTN KO C2C12 cells. Supplementary Figure S3: off-target analysis in mutant cloned cells. Three potential off-target sites were selected for sgRNA1. Sanger sequencing was performed to detect off-target mutagenesis using genomic DNA from mutant cells. PAM sequences are labelled in yellow. Base substitutions are shown in red. Supplementary Figure S4: bioinformatics analysis of increased or decreased proteins in C2C12 cells. Proteins with significantly altered accumulation after MSTN gene knockout in C2C12 cells are shown in red. Oxidative phosphorylation. The KEGG identifier and abbreviation key are provided in the Supporting Information. Supplementary Figure S5: bioinformatics analysis of present or absent proteins in C2C12 cells. Proteins with significantly altered accumulation after MSTN gene knockout in C2C12 cells are shown in red. (A) FoxO signaling pathway, (B) PPAR signaling pathway, (C) PI3K-AKT signaling pathway, (D) JAK-STAT signaling pathway. Supplementary Table S1: list of the top 20 upregulated and top 20 downregulated proteins. Supplementary Table S2: proteins associated with skeletal muscle cell development, fatty acid metabolism, the immune system, and mitochondrial energy metabolism. Supplementary Table S3: list of different bioinformatic methods for study changes in genome or proteome of MSTN KO.
