# Supplementary material for: Label-Free LC-MS/MS Proteomics Analyses Reveal Proteomic Changes Accompanying MSTN KO in C2C12 Cells
Source: Biomed Res Int. 2019 Apr 3;2019:7052456. doi: 10.1155/2019/7052456 (PMC6470438; doi:10.1155/2019/7052456)
Supplement: Supplementary Materials — Supplementary Figure S1: detection of MSTN CRISPR/Cas9-mediated targeting in C2C12 cells. (A) Mutation detection in C2C12 cells by T7EI cleavage assay. #3, #11, and #20 represent different MSTN KO cell clones. M: marker; WT: wild-type PCR products from C2C12 cells that were not treated with CRISPR/Cas9. (B) Sequences of modified MSTN alleles. Insertions are in red; deletions (-), insertions (+) shown to the right of each allele. Supplementary Figure S2: analysis of MSTN gene and protein expression in C2C12 myoblasts. (A) Relative expression level of MSTN was determined by qRT-PCR. (B) Western blot analysis of myostatin protein from NC and MSTN KO C2C12 cells. Supplementary Figure S3: off-target analysis in mutant cloned cells. Three potential off-target sites were selected for sgRNA1. Sanger sequencing was performed to detect off-target mutagenesis using genomic DNA from mutant cells. PAM sequences are labelled in yellow. Base substitutions are shown in red. Supplementary Figure S4: bioinformatics analysis of increased or decreased proteins in C2C12 cells. Proteins with significantly altered accumulation after MSTN gene knockout in C2C12 cells are shown in red. Oxidative phosphorylation. The KEGG identifier and abbreviation key are provided in the Supporting Information. Supplementary Figure S5: bioinformatics analysis of present or absent proteins in C2C12 cells. Proteins with significantly altered accumulation after MSTN gene knockout in C2C12 cells are shown in red. (A) FoxO signaling pathway, (B) PPAR signaling pathway, (C) PI3K-AKT signaling pathway, (D) JAK-STAT signaling pathway. Supplementary Table S1: list of the top 20 upregulated and top 20 downregulated proteins. Supplementary Table S2: proteins associated with skeletal muscle cell development, fatty acid metabolism, the immune system, and mitochondrial energy metabolism. Supplementary Table S3: list of different bioinformatic methods for study changes in genome or proteome of MSTN KO. [file 7052456.f1.docx]

Supporting Information

Supplementary Figure S1. Detection of MSTN CRISPR/Cas9-mediated targeting in C2C12 cells. (A) Mutation detection in C2C12 cells by T7EI cleavage assay. #3, #11, and #20 represent different *MSTN* KO cell clones. M: marker; WT: wild-type PCR products from C2C12 cells that were not treated with CRISPR/Cas9. (B) Sequences of modified *MSTN* alleles. Insertions are in red; deletions (-), insertions (+) shown to the right of each allele.

Supplementary Figure S2. Analysis of *MSTN* gene and protein expression in C2C12 myoblasts. (A) Relative expression level of MSTN was determined by qRT-PCR. (B) Western blot analysis of myostatin protein from NC and MSTN KO C2C12 cells.

Supplementary Figure S3. Off-target analysis in mutant cloned cells. Three potential off-target sites were selected for sgRNA1. Sanger sequencing was performed to detect off-target mutagenesis using genomic DNA from mutant cells. PAM sequences are labelled in yellow. Base substitutions are shown in red.

Supplementary Figure S4. Bioinformatics analysis of increased or decreased proteins in C2C12 cells. Proteins with significantly altered accumulation after MSTN gene knockout in C2C12 cells are shown in red. Oxidative phosphorylation. The KEGG identifier and abbreviation key are provided in the Supporting Information.

Supplementary Figure S5. Bioinformatics analysis of present or absent proteins in C2C12 cells. Proteins with significantly altered accumulation after MSTN gene knockout in C2C12 cells are shown in red. (A) FoxO signalling pathway, (B) PPAR signalling pathway, (C) PI3K-AKT signalling pathway, (D) JAK-STAT signalling pathway.

Supplementary Table S1. List of the top 20 upregulated and top 20 downregulated proteins.

Supplementary Table. S2. Proteins associated with skeletal muscle cell development, fatty acid metabolism, the immune system and mitochondrial energy metabolism.

Supplementary Table. S3. List of different bioinformatic methods for study changes in genome or proteome of *MSTN* KO

Supplementary material


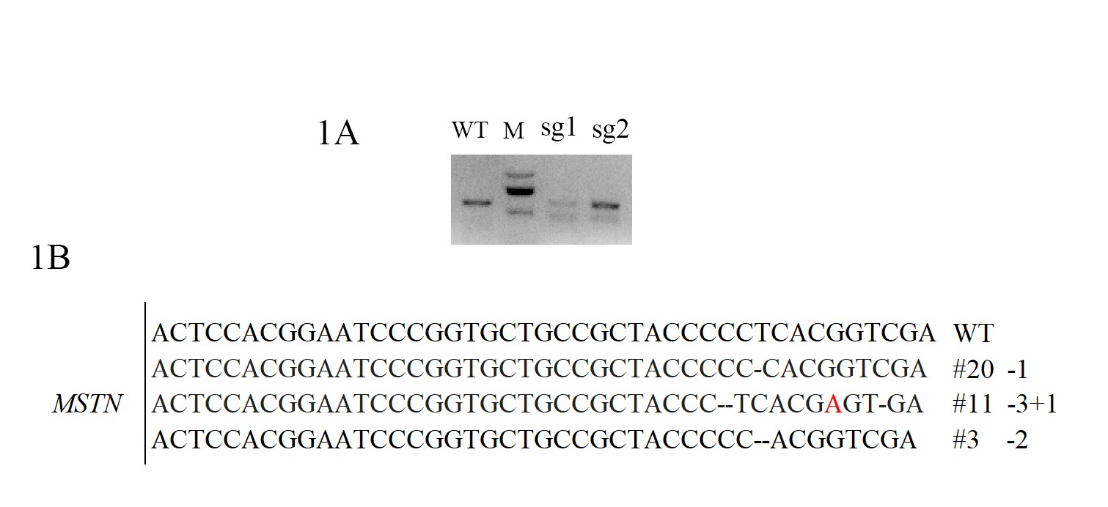


Supplementary Figure S1. Detection of MSTN CRISPR/Cas9-mediated targeting in C2C12 cells. (A) Mutation detection in C2C12 cells by T7EI cleavage assay. #3, #11, and #20 represent different *MSTN* KO cell clones. M: marker; WT: wild-type PCR products from C2C12 cells that were not treated with CRISPR/Cas9. (B) Sequences of modified *MSTN* alleles. Insertions are in red; deletions (-), insertions (+) shown to the right of each allele.

|  | sgRNA_sq | chr' | pos | target_sq | strain | mismatch |
| --- | --- | --- | --- | --- | --- | --- |
| sgRNA1 | AGGCTTCAAAATCGACCGTGAGG | chromosome 1 | 53066357 | AGGCTTCAAAATCGACCGTGAGG | - | 0 |
| OTS | AGGCTTCAAAATCGACCGTGAGG | chromosome 17 | 75489081 | AGGCTTTAAAATGGACAGTGAGG | - | 3 |


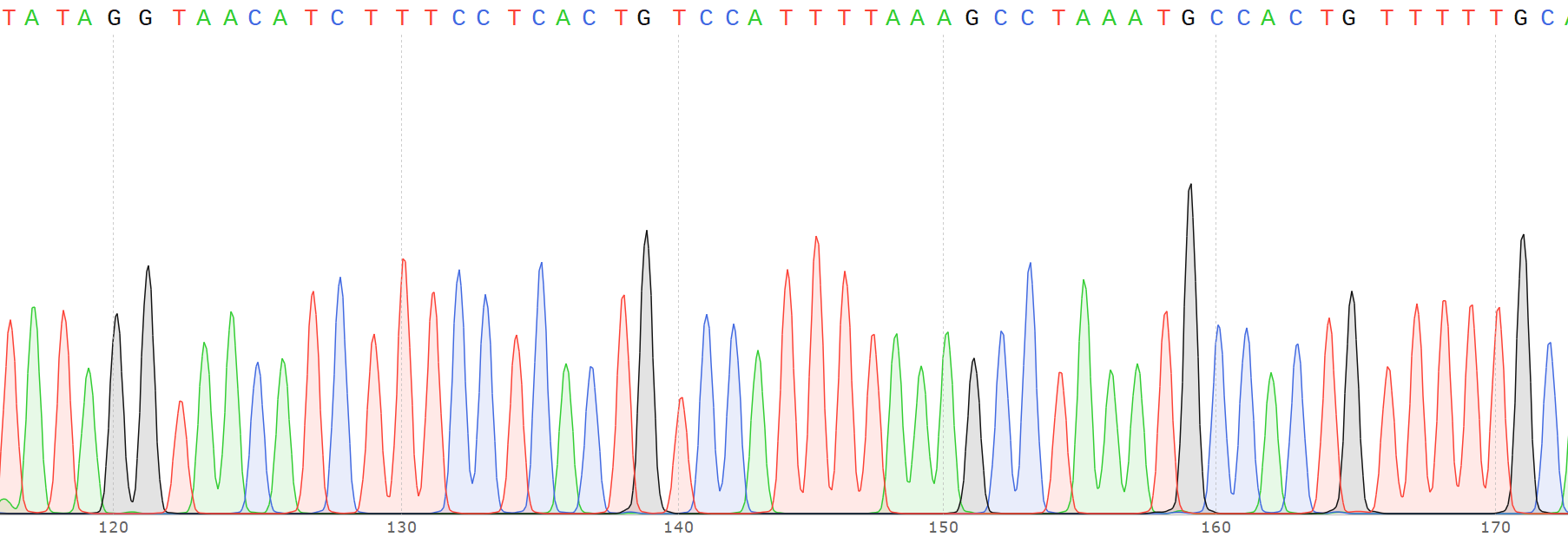


Supplementary Figure S2. Off-target analysis in mutant cloned cells. Three potential off-target sites were selected for sgRNA1. Sanger sequencing was performed to detect off-target mutagenesis using genomic DNA of mutant cells. PAM sequences are labelled in green. Base substitutions are shown in red, (OTS, off-target site).


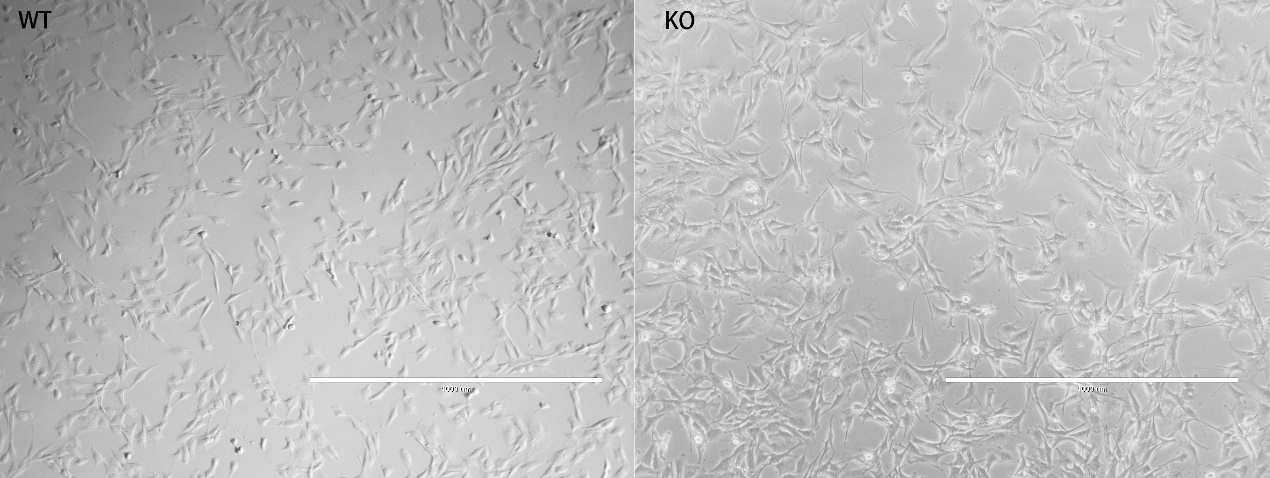


**A**


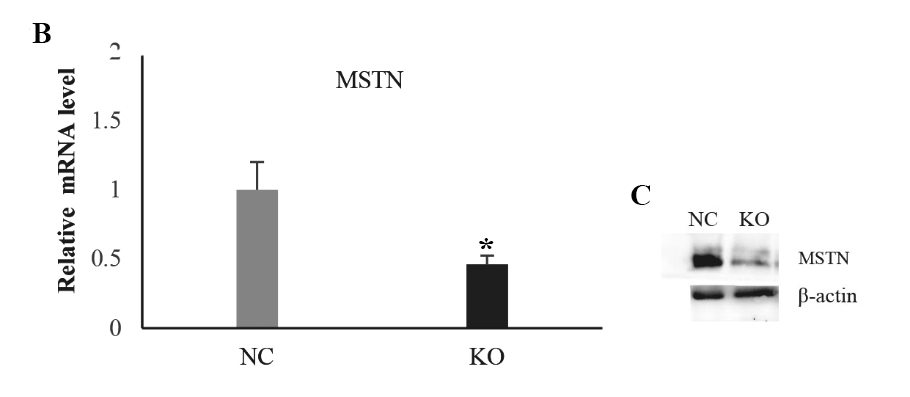


Supplementary Figure S3. Analysis of *MSTN* gene and protein expression in C2C12 myoblasts. (A) Electron micrograph of *Mstn* KO and wild type C2C12 cells. (B) Relative expression level of MSTN was determined by qRT-PCR. (C) Western blot analysis of myostatin protein from NC and MSTN KO C2C12 cells.


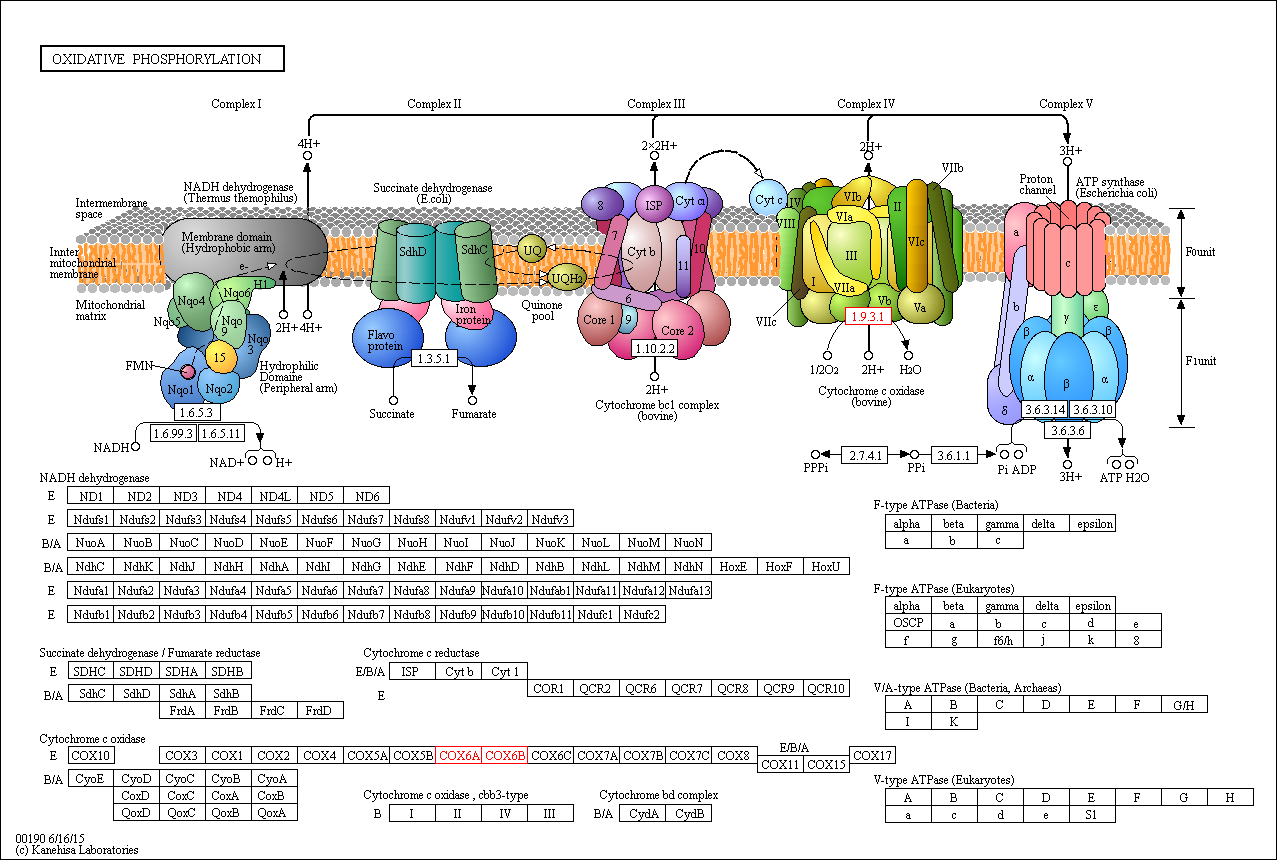


Supplementary Figure S4. Bioinformatics analysis of increase or decrease proteins in C2C12 cells. Proteins with significantly altered accumulation after MSTN gene knockout in C2C12 cells are shown in red. Oxidative phosphorylation. The KEGG identifier and abbreviation key are provided in the Supporting Information.


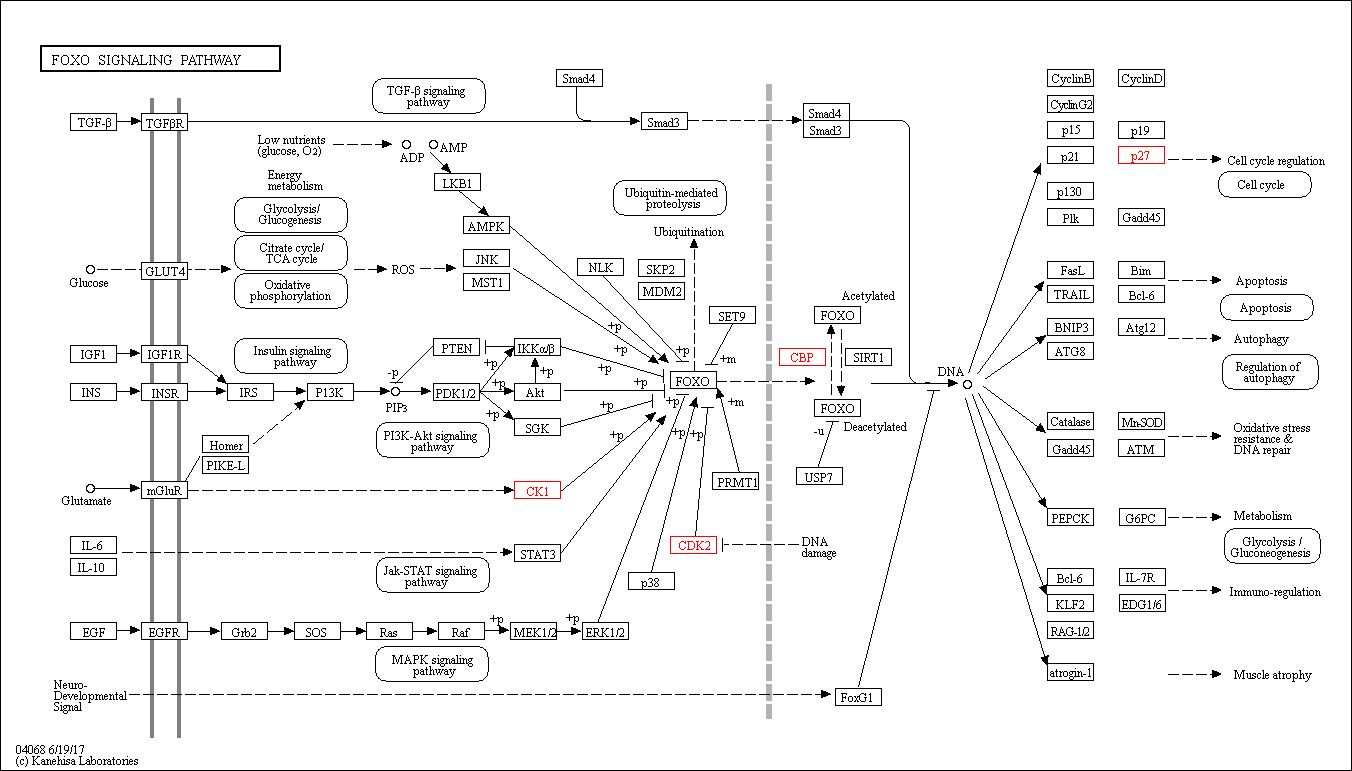


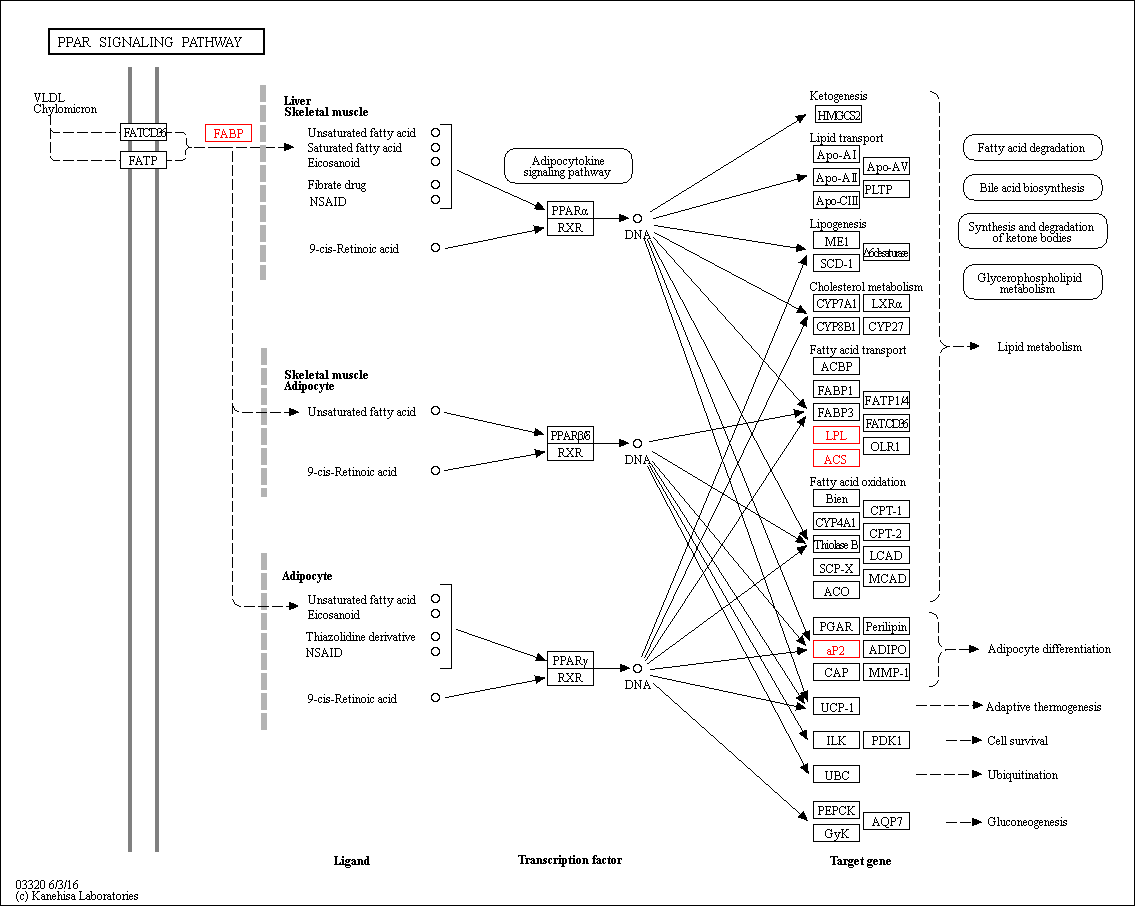


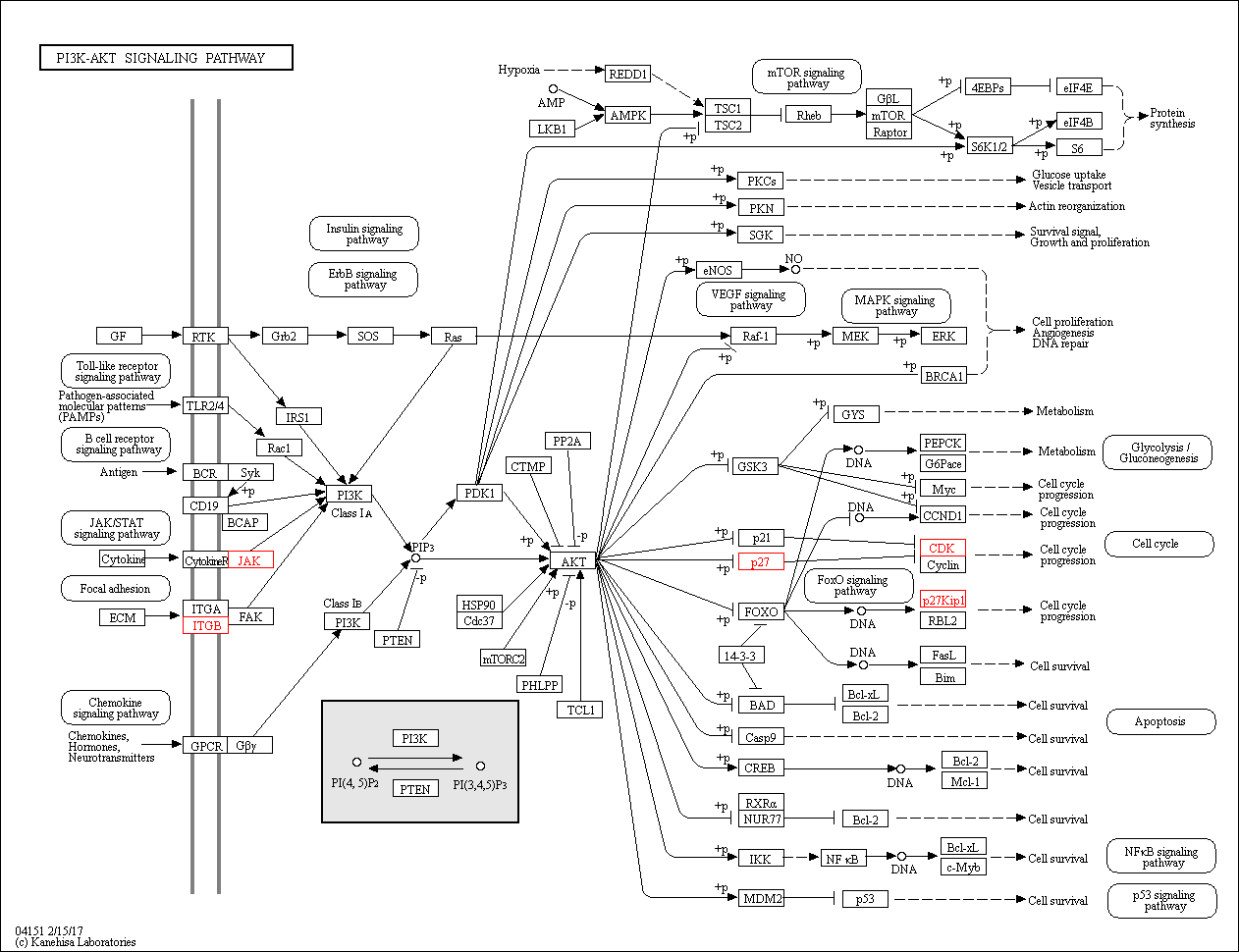


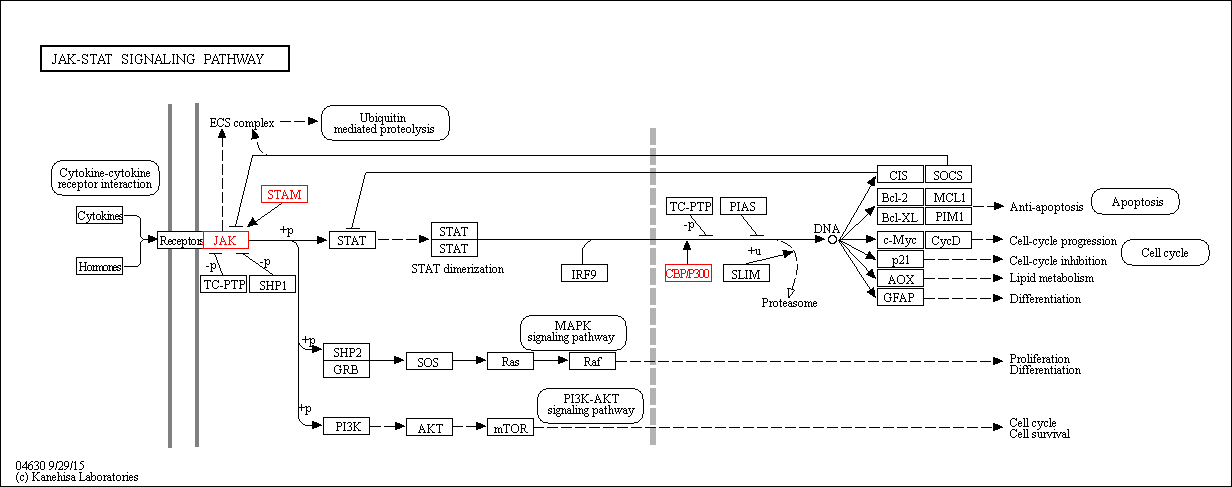


Supplementary Figure S5. Bioinformatics analysis of present *or absent* proteins in C2C12 cells. Proteins with significantly altered accumulation after MSTN gene knockout in C2C12 cells are shown in red. (A) FoxO signalling pathway, (B) PPAR signalling pathway, (C) PI3K-AKT signalling pathway, (D) JAK-STAT signalling pathway.

Supplementary Table S1. c top 20 upregulated and top 20 downregulated proteins.

| Protein IDs | Description | Gene name | Unique  peptides | MW [kDa] | *p*-value | Treat/control |
| --- | --- | --- | --- | --- | --- | --- |
| E9PWK1 | Epoxide hydrolase | Ephx1 | 21 | 51.0 | 0.002 | 2.4 |
| G5E8R1 | tropomyosin 1 | Tpm1 | 0 | 28.6 | 0.002 | 2.1 |
| P52760 | 2-iminobutanoate/2-iminopropanoate deaminase | Rida | 4 | 14.3 | 0.002 | 5.5 |
| A2RSS6 | Aspartylglucosaminidase | Aga | 5 | 37.0 | 0.004 | 2.2 |
| Q58D62 | Fetuin-B | FETUB | 9 | 42.7 | 0.007 | 2.0 |
| Q6P8U3 | Mitochondrial ribosomal protein L19 | Mrpl19 | 1 | 33.5 | 0.007 | 4.0 |
| Q3UAS4 | Uncharacterized protein | Psap | 15 | 60.0 | 0.011 | 2.3 |
| Q9R0Y5 | Adenylate kinase isoenzyme 1 | Ak | 11 | 21.5 | 0.012 | 2.3 |
| Q3SZH5 | Angiotensinogen | AGT | 7 | 45.5 | 0.014 | 2.8 |
| G5E850 | Cytochrome b-5, isoform CRA_a | Cyb5a | 5 | 11.1 | 0.014 | 2.1 |
| E9QLJ0 | Cardiomyopathy-associated protein 5 | Cmya5 | 2 | 405.6 | 0.014 | 2.5 |
| Q3UGR5 | Haloacid dehalogenase-like hydrolase domain-containing protein 2 | Hdhd2 | 1 | 28.7 | 0.017 | 2.6 |
| P28800 | Alpha-2-antiplasmin, serine protease inhibitor. | SERNF | 8 | 54.7 | 0.020 | 3.1 |
| Q2UVX4 | Complement C3 | C3 | 11 | 187.4 | 0.021 | 2.4 |
| Q3V2R9 | Uncharacterized protein | Acadsb | 3 | 37.7 | 0.022 | 2.0 |
| G3X922 | DnaJ heat shock protein family (Hsp40) member C13 | Dnajc3 | 14 | 254.4 | 0.023 | 2.4 |
| Q3MHN5 | Vitamin D-binding protein, Involved in vitamin D transport and storage, scavenging of extracellular G-actin. | GC | 9 | 53.3 | 0.023 | 2.1 |
| Q9R118 | Serine protease HTRA1, HTRA1-generated fibronectin fragments further induce synovial cells to upregulate MMP1 and MMP3 production. | Htra1 | 11 | 51.2 | 0.025 | 3.0 |
| Q9DCW5 | Cytochrome c oxidase subunit 6A, mitochondrial, | Cox6a1 | 1 | 12.5 | 0.025 | 4.3 |
| P01966 | Hemoglobin subunit alpha, Involved in oxygen transport from the lung to the various peripheral tissues. | HBA | 7 | 15.2 | 0.026 | 2.3 |
|  |  |  |  |  |  |  |
| Q4JG03 | Mcl-1 ubiquitin ligase | Huwe1 | 20 | 482.7 | 0.002 | 0.4 |
| F6QL70 | 60S ribosomal protein L29 | Gm17669 | 4 | 17.0 | 0.002 | 0.4 |
| Q62219 | Transforming growth factor beta-1-induced transcript 1 protein, functions as a molecular adapter coordinating multiple protein-protein interactions at the focal adhesion complex and in the nucleus. | Tgfb1i1 | 9 | 50.1 | 0.003 | 0.3 |
| Q6DID7 | Protein wntless homolog, regulates Wnt protein sorting and secretion in a feedback regulatory mechanism. | Wls | 9 | 62.2 | 0.004 | 0.2 |
| Q8BTJ1 | Phosphoserine aminotransferase, this protein is involved in step **2** of the subpathway that synthesizes L-serine from 3-phospho-D-glycerate. | Psat1 | 15 | 40.5 | 0.004 | 0.4 |
| P56391 | Cytochrome c oxidase subunit 6B1, connects the two COX monomers into the physiological dimeric form. | Cox6b1 | 4 | 10.1 | 0.004 | 0.2 |
| Q3TJN1 | Branched-chain-amino-acid aminotransferase | Bcat1 | 7 | 42.8 | 0.004 | 0.2 |
| Q04857 | Collagen alpha-1(VI) chain, collagen VI acts as a cell-binding protein. | Col6a1 | 18 | 108.5 | 0.009 | 0.3 |
| B9EJ54 | MCG21756, isoform CRA_b | Nup205 | 22 | 227.5 | 0.009 | 0.3 |
| Q9QXE7 | F-box-like/WD repeat-containing protein TBL1X, F-box-like protein involved in the recruitment of the ubiquitin/19S proteasome complex to nuclear receptor-regulated transcription units. | Tbl1x | 2 | 56.8 | 0.009 | 0.4 |
| Q8BLY2 | Probable threonine-tRNA ligase 2, cytoplasmic, | Tarsl2 | 3 | 91.3 | 0.011 | 0.3 |
| F2WWK6 | Delta-6 desaturase | Fads2 | 4 | 51.4 | 0.011 | 0.4 |
| Q5M9K3 | MCG23116, isoform CRA_a | Uba52 | 2 | 14.7 | 0.011 | 0.3 |
| B2RQQ5 | Microtubule-associated protein 1B, | Map1b | 38 | 270.3 | 0.011 | 0.4 |
| Q3TX57 | Procollagen, type I, alpha 2, [transforming growth factor beta receptor signalling pathway](https://www.ebi.ac.uk/QuickGO/term/GO:0007179), [skeletal system development](https://www.ebi.ac.uk/QuickGO/term/GO:0001501). | Col1a2 | 28 | 129.6 | 0.012 | 0.1 |
| Q80U93 | Nuclear pore complex protein Nup214, May serve as a docking site in the receptor-mediated import of substrates across the nuclear pore complex. | Nup214 | 11 | 213.0 | 0.013 | 0.4 |
| Q8BK29 | Uncharacterized protein | Prpsap1 | 6 | 39.4 | 0.013 | 0.4 |
| Q9CQW9 | Interferon-induced transmembrane protein 3, [negative regulation of cell proliferation](https://www.ebi.ac.uk/QuickGO/term/GO:0008285). | Ifitm3 | 3 | 15.0 | 0.014 | 0.3 |
| P07742 | Ribonucleoside-diphosphate reductase large subunit, provides the precursors necessary for DNA synthesis. Catalyses the biosynthesis of deoxyribonucleotides from the corresponding ribonucleotides. | Rrm1 | 13 | 90.2 | 0.017 | 0.1 |
| P02768-1 | Serum albumin, its main function is the regulation of the colloidal osmotic pressure of blood. Major zinc transporter in plasma, typically binds approximately 80% of all plasma zinc. | ALB | 2 | 69.4 | 0.017 | 0.3 |

Supplementary Table. S2. Proteins associated with skeletal muscle cell development, fatty acid metabolism, the immune system and mitochondrial energy metabolism.

| Pathway Name | Protein Name | Gene name |
| --- | --- | --- |
| Skeletal muscle cell development | tropomyosin 1 | Tpm1 |
|  | serine protease HTRA1 | HTRA1 |
|  | microtubule-associated protein 1B | Map 1b |
|  | myosin-9 | Myf9 |
|  | cytoskeleton-associated protein 5 | Ckap5 |
|  | follistatin-related protein 1 | Fst1 |
|  | filamin-C | Flnc |
|  | integrin beta-1 | ITGB1 |
|  | TRIO and F-actin-binding protein | Triobp |
|  | microtubule-associated protein 1 light chain 3 beta | Map1lc3b |
| Fatty acid metabolism | fatty acid synthase (FAS) | FAS |
|  | delta-6 desaturase | Fads2 |
|  | succinate-CoA ligase subunit beta | Sucla2 |
|  | acetyl-CoA acetyltransferase | Acat2 |
| Immune system process | CD109 | CD109 |
|  | CD151 | CD151 |
|  | Synaptosomal-associated protein 29 | SNAP29 |
|  | Uncharacterized protein | Ctsb |
|  | Cathepsin D | Ctsd |
|  | Vascular cell adhesion protein 1 | Vcam1 |
| Mitochondrial energy metabolism | mitochondrial ribosomal protein L19 | Mrpl9 |
|  | mitochondrial import inner membrane translocase subunit Tim23 | Gm10273 |
|  | Cytochrome b-5 | Cyb5 |
|  | Cytochrome c oxidase subunit 6A | Cox6a1 |
|  | ATP synthase subunit d | Gm10250 |
|  | gluose-6-phosphate 1-dehydrogease | G6pdx |
| TGF-beta signalling pathway | Rho guanine nucleotide exchange | GEF-H1 |
|  | CDC42 GTPase-activating protein | Arhgap31 |
|  | Rac1 |  |
|  | Serine/threonine-protein kinase PAK2 | PAK2 |
|  | Ras family member A | Rho A |
|  | CD109 |  |
| ECM-receptor interaction | Collagen alpha-1(VI) chain | Col6a1 |
|  | Procollagen, type I, alpha 2 | Col1a2 |
|  | Integrin beta | Itgb5 |
|  | collagen, type I, alpha | COL1A |
| MAPK signalling pathway | transducin (beta)-like 1 | TBL1 |
|  | SHC-transforming protein 1 | SHC1 |

Supplementary Table. S3. List of different bioinformatic methods for study changes in genome or proteome of *MSTN* KO

| Experiment name | Sample-model | Bioinformatic results | method |
| --- | --- | --- | --- |
| RNA-seq | *MSTN*-KO goat/WT goat | Actin cytoskeleton, fatty acid metabolic system, energy metabolic, Oxidative phosphorylation and immune system | The counts of the read numbers mapped to each gene were processed by HTSeq v0.6.1, and the FPKM. Differentially expressed genes (DEGs) were identified using the R package, DEGs were determined by DEGseq with a cutoff threshold of *P*-value *<* 0.05. |
| 2-dimensional electrophoresis (2DE) | *MSTN*-null mice/WT mice | Aldehyde reductase were up-regulated | Proteins of Mr＞90 kDa were observed and proteins of a low mass were not well resolved. |
| High-resolution mass spectrometry couple with SILAC mouse technology | *MSTN*-knockout (*MSTN*-/-) mice/anti-*MSTN* antibody mice/WT mice | Myosin heavy chain, oxidative phosphorylation, mitochondrial and catabolic processes | 8 ppm for MS tolerance, 0.8 Da for MS/MS tolerance and 1 missed cleavage, identified peptides were filtered using 1% false discovery rate ( FDR), differentially expressed proteins were filtered (*p*-value ＞1.5 or ＜0.67). |
| Label-free LC-MS/MS | *MSTN*-KO C2C12 cell/empty vector control C2C12 cell | Actin cytoskeleton, fatty acid metabolic system, energy metabolic, Oxidative phosphorylation, immune system, mitochondrial and catabolic processes | 6 ppm for MS tolerance, 2 missed cleavages, identified peptides and proteins were filtered using ≤1% false discovery rate ( FDR), Razor and unique peptides were used for protein quantification, differentially expressed proteins were filtered (*p*-value ≥ 2 or ≤ 0.5). |
